# Pericytes in the brain and heart: functional roles and response to ischaemia and reperfusion

**DOI:** 10.1093/cvr/cvae147

**Published:** 2024-07-29

**Authors:** Turgay Dalkara, Leif Østergaard, Gerd Heusch, David Attwell

**Affiliations:** Department of Neuroscience, Bilkent University, Ankara 06800 Türkiye; Department of Molecular Biology and Genetics, Bilkent University, Ankara 06800 Türkiye; Center of Functionally Integrative Neuroscience (CFIN), Department of Clinical Medicine, Aarhus University, Aarhus, Denmark; Institute for Pathophysiology, West German Heart and Vascular Center, University of Duisburg-Essen, Essen, Germany; Department of Neuroscience, Physiology & Pharmacology, University College London, Gower St., London WC1E 6BT, UK

**Keywords:** Stroke, AMI, No-reflow, Pericytes, Microcirculation

## Abstract

In the last 20 years, there has been a revolution in our understanding of how blood flow is regulated in many tissues. Whereas it used to be thought that essentially all blood flow control occurred at the arteriole level, it is now recognized that control of capillary blood flow by contractile pericytes plays a key role both in regulating blood flow physiologically and in reducing it in clinically relevant pathological conditions. In this article, we compare and contrast how brain and cardiac pericytes regulate cerebral and coronary blood flow, focusing mainly on the pathological events of cerebral and cardiac ischaemia. The cerebral and coronary capillary beds differ dramatically in morphology, yet in both cases, pericyte-mediated capillary constriction plays a key role in restricting blood flow after ischaemia and possibly in other pathological conditions. We conclude with suggestions for therapeutic approaches to relaxing pericytes, which may prove useful in the long-term for reducing pericyte-induced ischaemia.

**This article is part of the Spotlight issue on brain, heart, and vessels crosstalk.**

## 1. Introduction

A Nobel Prize was given in 1920 for the concept of regulation of tissue blood flow at the capillary level by contractile pericytes.^1^ Despite this, the concept of capillary regulation of blood flow was later largely lost from the literature, as cardiovascular physiologists focused more on autoregulation of perfusion, until the pioneering work of Puro^2^ on retinal pericytes re-invigorated this idea at the start of this century. Subsequent work has shown for the brain and retina that pericytes not only play a role in increasing microcirculatory blood flow when neurons are active,^2,3^ they also restrict blood flow after ischaemia,^3,4^ in Alzheimer’s disease,^5^ in hyperoxia,^6^ and possibly in COVID-19.^7^ In the pancreas, heart, and kidney, pericytes are suggested to regulate blood flow,^8–10^ and in the heart and the kidney, pericyte-mediated capillary constriction reduces blood flow after ischaemia.^11–13^

In view of the increasing evidence for both physiological and pathological regulation of tissue blood flow by pericytes, it is of interest to document the similarities of and differences between pericyte-mediated control of blood flow in different organs. Here we review these aspects of blood flow control for the brain and the heart, which have strikingly different topology to their vascular beds, yet which both exhibit control of blood flow by pericytes in health and disease.

## 2. Pericyte phenotype/genotype along the arteriovenous axis

**Figure 1:**
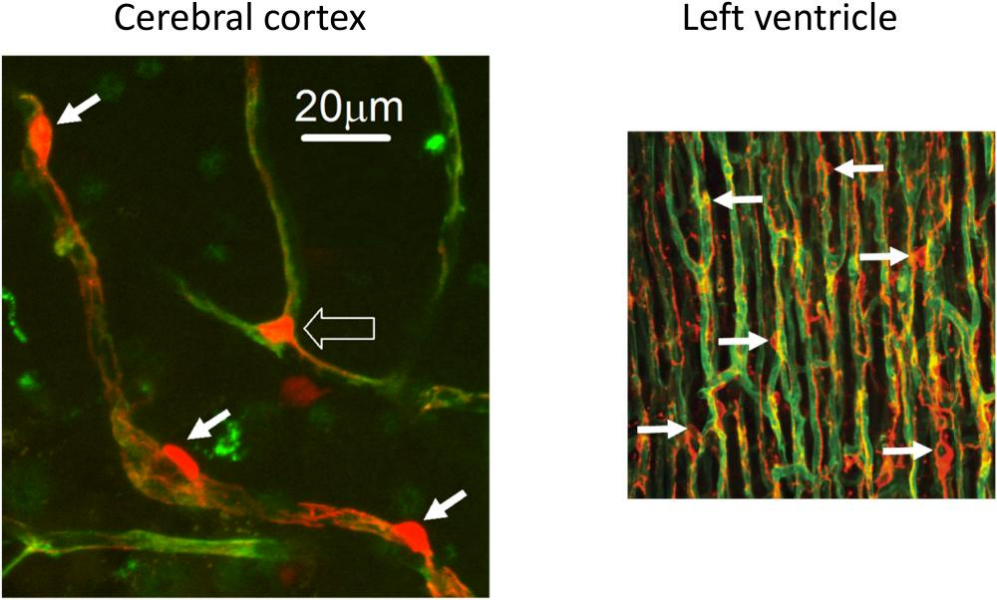
Pericytes in the cerebral cortex and cardiac ventricle. Capillaries labelled with FITC-conjugated IB4 (green, which labels basement membrane) and pericytes labelled with antibody to NG2 (red) in the cerebral cortex (left) and cardiac ventricle (right). Capillary bed in the cerebral cortex consists of branches off of penetrating arterioles that then branch ∼6 times in rodents, before merging again to join the ascending venule; dark spaces are occupied by neurons and glia. In contrast, the denser vascular bed of the ventricle consists largely of parallel capillaries surrounding individual myocytes (dark spaces). Solid white arrows label pericyte somata. Open arrow labels a pericyte at a capillary branch point. Images taken by Catherine Hall and Fergus O’Farrell in the Attwell lab for Hall *et al*.^3^ and O'Farrell *et al*.^11^ Scale bar applies to both panels.

Pericyte morphology varies along the arteriovenous axis and also between capillary beds in different tissues^11,13–15^ (*Figure 1*). Pericytes are found embedded within two layers of the basement membrane of the microvascular wall. They can have a soma protruding out of the vessel wall, the so-called bump-on-a-log morphology, which is seen for pericytes in the middle of capillary segments, or have a soma at the branch point of capillaries, e.g. touching the incoming and one of the outgoing capillaries. They extend processes along and around capillaries, but to varying extents depending on position in the vascular bed. At the arteriolar end of the cerebral microcirculation, pericyte processes almost completely enwrap the endothelial tube circumferentially, and so are named ensheathing type pericytes.^15^ They are contractile and exhibit high α-SMA expression (see below). On downstream second–fourth-order capillary branches, pericyte processes become shorter with less complete coverage, giving a ‘mesh-like’ appearance, and these are designated as mesh type pericytes. Further downstream, mesh pericytes become limited to bifurcation points, whereas strand-like pericytes are located along the capillary. Their soma is situated in the middle of capillary segments and extends thin-strand-like processes helically enwrapping the vessel lumen. Accordingly, these pericytes are called thin-strand-like or helical or mid-capillary pericytes. They express relatively little α-SMA.

scRNAseq studies of cerebral pericytes have shown remarkable variation in the supposed marker genes for pericytes, probably reflecting variation of cell properties along the arteriovenous axis. For example, a comparison^16^ of five scRNAseq studies surveying 260 genes identified in mural cells found only three transcripts in common within the five studies (two genes related to myosin-based contraction and a folate transporter). Subsequent work by the Betsholtz group clustered cell types apparently using a criterion that pericytes express no α-smooth muscle actin (α-SMA),^17^ and thus concluded that all pericytes were transcriptomically identical, did not express α-SMA, and cannot contract. This is not consistent with the publication of numerous movies of pericytes contracting, especially at the arteriole end of the capillary bed (see next section), and the known arteriovenous gradient of pericyte expression of α-SMA (see below). Subsequent studies, using different clustering approaches, concluded there are several transcriptomically distinct classes of pericyte,^18,19^ which may in part correspond to position in the vascular bed, or to different cell classes that regulate solute transport and extracellular matrix organization. Importantly, in addition to differences of expression level of contractile proteins between different classes of pericytes and between pericytes and vascular smooth muscle cells (vSMCs), the transcriptomes of vSMCs and pericytes show striking differences in many genes,^16,20–22^ such as potassium inwardly rectifying channel subfamily J member 8 (Kcnj8), ATP binding cassette subfamily C member 9 (Abcc9), vitronectin (vtn), gamma-glutamyltransferase 1 (Ggt1), and interferon-induced transmembrane protein 1 (Ifitm1).

The capillary bed in the cardiac ventricles differs dramatically from that in the cerebral cortex (*Figure 1*), with most capillaries being parallel and only occasional short perpendicular capillaries linking them. The mean distance between pericytes in the adult rat heart^11^ (∼60 μm) is similar to that in the P21 rat cerebral cortex^3^ (∼45 μm). As reviewed below, pericytes in both tissues regulate blood flow, restricting it notably in pathological conditions. The different topology of the vascular beds in these tissues may result in pericytes in different locations being differentially important in controlling blood flow. For example, in the brain capillary bed, a simple mathematical argument demonstrates that pericytes on the first few branch orders from a penetrating arteriole will have most power to regulate flow,^23^ whereas in the heart, if there were no interconnecting ‘linker’ capillaries between adjacent straight capillaries, then contraction of a single pericyte anywhere on a straight capillary would be sufficient to reduce blood flow to the whole volume supplied by that vessel.

In addition to their role in the regulation of microcirculatory blood flow, pericytes contribute to the integrity of the blood–brain barrier by inducing the expression of tight junctions and suppressing transcytosis,^24–26^ and influence the passage of inflammatory cells across the vessel wall. Pericyte deficiency decreases the rise of blood flow evoked by neuronal activity,^27^ and causes blood–brain barrier breakdown and leakage of serum proteins and several neurotoxic macromolecules, leading to secondary neuronal degenerative changes.^24,26^ They also contribute to synthesis of extracellular matrix proteins and play a pivotal role in angiogenesis, and a sub-type of pericyte has been suggested to generate the fibrotic scar after CNS injury^28^ although more recent work has attributed this to perivascular fibroblasts.^29^ It is currently uncertain to what extent pericytes at different locations along the AV axis differentially impose a tight blood–brain barrier. Similarly, the extent to which cardiac pericytes influence the properties of the endothelial cell layer in coronary capillaries remains to be studied in depth.

## 3. Identifying pericytes

Pericytes are often labelled, transgenically or with antibodies, based on their expression of the proteoglycan NG2 (gene *Cspg*), which is also expressed by oligodendrocyte precursor cells (OPCs) but pericytes and OPCs can be distinguished by their expression of the growth factor receptors PDGFRβ and PDGFRα, respectively. As well as NG2, pericytes are often defined by labelling for PDGFRβ, but both NG2 and PDGFRβ are also expressed by vascular smooth muscle cells (SMCs). The fundamental distinction between arteriolar SMCs and pericytes is morphological, in that SMCs form a contiguous layer around arterioles, with circumferential processes abutting each other, while pericyte somata are spatially isolated from each other (typically with a separation of 50–160 μm). Pericytes extend processes along and around the capillary, with a morphology that depends on arteriovenous position in the capillary bed (with more circumferential processes at the arteriolar end of the bed).^15^ Perivascular fibroblasts are found near arterioles and the first few branches of the capillary bed. They differ from pericytes in that pericytes are totally enveloped by the basement membrane (which can be labelled by the lectin IB4) while fibroblasts are outside the basement membrane and only contact it on one side, and fibroblasts (but not pericytes) also express PDGFRα^30,31^

## 4. Pericytes are contractile cells

Since their initial identification by Eberth in Germany and Rouget in France, pericytes have been proposed to have contractile capability and play a role in regulating microcirculatory blood flow.^1^ For the brain, after controversies over decades, it is now widely accepted that blood flow in the microcirculatory network is regulated by contractile pericytes present on the first four branch orders (counting from a penetrating arteriole) of the capillary bed. Direct imaging of capillary diameter being adjusted by pericytes has been reported both for *ex vivo* retinal and brain slice preparations in response to exogenous depolarization or neurotransmitter application,^14^ and *in vivo* in cerebral cortex.^3^ The crucial role of pericytes is demonstrated by the fact that depolarizing parts of the capillary wall where there are no pericytes do not evoke constriction,^14^ and the fact that pericyte contraction tends to produce the largest capillary constriction near the pericyte soma, where circumferential processes of the pericyte (that mediate constriction when they contract) are mainly located.^5^

For the arteriolar end of the brain capillary bed, multiple lines of recent evidence have confirmed the *in vivo* contractile nature of the pericytes, akin to vascular smooth muscle cells (vSMCs), as originally suggested. Pericytes situated over the first- to fourth-order capillaries exhibit substantial luminal coverage with their often-circumferential processes, and express α-smooth muscle actin (α-SMA), consistent with a role in regulating blood flow through contraction or relaxation. Although there is not a consensus, these cells are named ‘contractile’ or ‘ensheathing’ pericytes and likely correspond to ‘hybrid’ cells, a term used in the past to emphasize that they exhibit a transitional morphology between SMCs upstream and mid-capillary bed pericytes downstream that exhibit more longitudinal processes (termed ‘strand-like’: see below). As for vSMCs, their adjustment of capillary diameter is regulated by changes of intracellular calcium concentration.^5,14,32^

**Figure 2:**
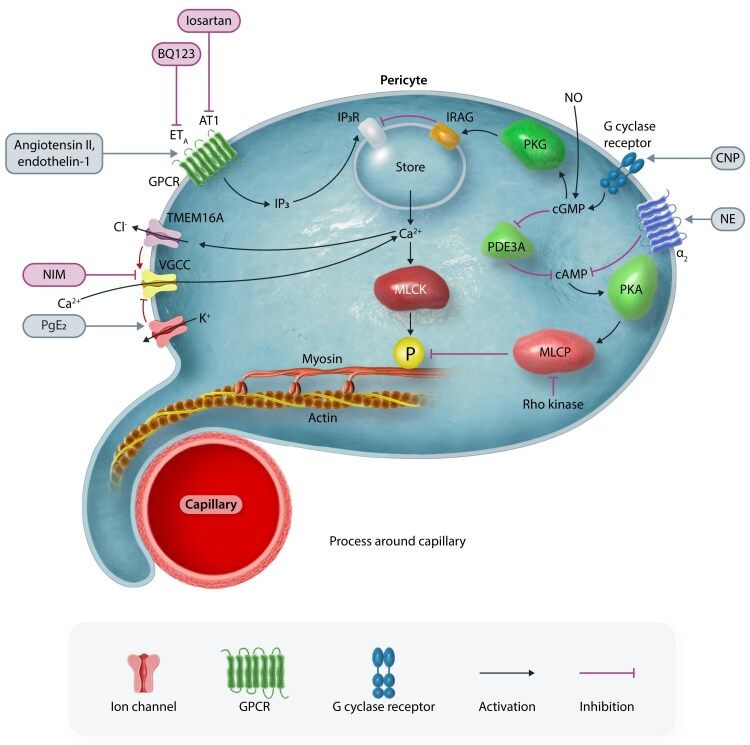
Some pathways regulating pericyte-mediated capillary constriction and dilation. Pericyte contraction (and hence capillary constriction) is mediated by myosin and actin, regulated by myosin light kinase (MLCK) phosphorylating myosin. When common constricting agents like endothelin-1 or angiotensin activate ET_A_ or AT1 G_q_-coupled receptors, they generate IP_3_ that releases Ca^2+^ from internal stores, to activate MLCK and contraction. This contraction is amplified^34^ by Ca^2+^ activating TMEM16A Cl^−^ channels, allowing Cl^−^ exit to generate a depolarization that activates voltage-gated Ca^2+^ channels (VGCC). Norepinephrine (NE) activates α_2_ receptors^36^ that lower [cAMP], thus resulting in protein kinase A activating myosin light chain phosphatase (MLCP) less, so that relaxation is inhibited and contraction evoked. Agonists stimulating Rho kinase similarly promote contraction (either by inhibiting MLCP or by extending actin filaments). PgE_2_ generated by arachidonic acid metabolism, e.g. in active neurons, can evoke dilation by raising [cAMP] and thus activating MLCK or by activating K^+^ channels^3^ to evoke a hyperpolarization that inhibits Ca^2+^ entry via VGCCs. Pericyte contraction might be opposed for therapeutic purposes by blocking vasoconstricting receptors (e.g. with BQ123 or losartan),^3^ blocking VGCCs with nimodipine,^34^ activating guanylate cyclase (G cyclase) receptors with C-type natriuretic peptide (CNP)^5^ to raise [cGMP] and [cAMP] thus (via IRAG) inhibiting Ca^2+^ release from stores and activating MLCK, or blocking Rho kinase.^13^ Endothelial or exogenously generated nitric oxide (NO) may also dilate by raising [cGMP], but physiologically this pathway appears to be active in SMCs but not in pericytes.^37^

Unlike the consensus on the contractility of upstream pericytes, there has been skepticism regarding the potential contractile role of mid-capillary pericytes on downstream (≥fourth order) capillaries. These pericytes, displaying morphological characteristics of a mesh or thin-strand nature (see below), have been suggested not to contribute significantly to blood flow regulation due to their thin processes (which spiral around the vessel in a less circumferential orientation than for the upstream pericytes), limited mural coverage and expression of little or no α-SMA. However, it has been demonstrated that the diameter of higher order capillaries is in fact adjustable^32^ and that optogenetic activation of the pericytes on these vessels leads to constriction^33^ even though this constriction is slower than that seen for lower branch order pericytes. It is likely that this skepticism originates from current technical limitations rather than an absence of contractility and α-SMA expression, as discussed below. Another possible contributing difference is that, while upstream capillary pericyte contraction is mediated by G protein coupled receptor evoked Ca^2+^ release from internal stores and Ca^2+^ entry via voltage-gated Ca^2+^ channels (amplified by the action of Ca^2+^-gated chloride channels that enhance pericyte depolarization),^34^ pericytes in the middle of the capillary bed have their [Ca^2+^]_i_ controlled independently of voltage-gated Ca^2+^ channels.^35^ Likely mechanisms controlling pericyte tone are summarized in *Figure 2*.

## 5. Substrates for contractility in mid-capillary bed pericytes

Mid-capillary bed pericytes were previously suggested not to regulate capillary diameter (a suggestion disproven by the optogenetic experiments described above) because α-SMA, the contractile actin isoform in vascular smooth muscle cells, shows a lower level in pericytes on capillaries downstream of the first three to four branch orders, both in the brain and retina. In contrast, its partner in the contractile apparatus, myosin II (predominantly the Myh11 isoform in pericytes) is detectable by immunohistochemistry even in mid-capillary bed pericytes. The contractility recently observed in mid-capillary bed pericytes (which may be harder to detect because of the small size of these vessels) may thus be mediated by small amounts of α-SMA, the presence of which can only be revealed using rapid tissue fixation techniques or toxins that inhibit actin depolymerization and/or promote actin polymerization^38,39^ (reviewed in Erdener *et al*.^40^). The small pool of α-SMA in mid-capillary bed pericytes, constantly de/re-polymerizing, is likely maintained by slow actin monomer turnover, consistent with the low α-SMA mRNA levels reported in these cells. Indeed, in the intermediate layer of the retina, seventh to eighth branch order capillaries displayed the greatest dilation during light stimulation. Mid-capillary bed pericytes may require only a small concentration of contractile proteins because the capillaries they surround have a lower intraluminal pressure than upstream pericytes.

Mid-capillary bed pericytes *in vivo* tend to show slower but longer lasting contractions than pericytes on the first few branch orders of the capillary bed,^33^ possibly reflecting the lower level of α-SMA, a less circumferential organization of their processes around the capillary, and the involvement of reactive oxygen species generation and activation of Rho kinase (*Figure 2*) in evoking the contraction.^33^ An accompanying decrease in capillary blood flow suggested that measured diameter changes were not artefactual.

There is a dynamic balance between filamentous F-actin and monomeric (globular) G-actin in cells. Hence, F-actin polymerization, a universal cellular mechanism providing contractility, motility, and tone, likely plays a role in pericyte contraction similar to its function in vascular smooth muscle cells.^41^ In mid-capillary bed pericytes, the rapid depolymerization of α-SMA precluding its detection suggests that polymerization may regulate the length of α-SMA filaments in these cells, consistent with the role of the Rho kinase—LIM kinase—cofilin pathway in contraction mentioned above. In addition, polymerization of cytoskeletal (non-contractile) β and γ actin filaments can function in parallel to actomyosin-mediated contraction by providing cellular stiffness against mechanical deformation during contraction, as is well documented in vSMCs.^42^ Cytoskeletal actin filaments also form a lattice between contracting stress fibres and the extracellular matrix, transforming tension developed by actomyosin cross bridge cycling into vessel constriction.^41,43^ This process is also thought to contribute to vSMC tone, as blocking actin polymerization induces vessel relaxation without affecting actomyosin-mediated contraction.^44^ Further research is needed to elucidate the relative contribution and timing of actomyosin- and polymerization-mediated contraction/relaxation mechanisms in pericyte contraction.

## 6. Activity-evoked dilation of capillaries in physiological conditions

Numerous mediators have been reported to increase cerebral and coronary blood flow in response to increases of neuronal or muscle activity.^45,46^ These include signalling molecules such as nitric oxide and the arachidonic acid derivatives prostaglandin E_2_ and I_2_, and metabolically driven messengers such as adenosine, pH, CO_2_, O_2_, and a local rise of [K^+^] concentration. In general, these agents work by activating K^+^ channels to hyperpolarize contractile mural cells and thus decrease voltage-gated Ca^2+^ entry, or by raising the intracellular concentration of cyclic AMP or GMP to either decrease Ca^2+^ release from intracellular stores or to activate myosin light chain phosphatase (MLCP) and decrease the sensitivity of the contractile filaments to a [Ca^2+^]_i_ rise.

In brain pericytes, neuronal activity evokes an outward current sufficient to hyperpolarize the pericyte by about 10 mV from its resting potential of −48 mV, thus closing voltage-gated Ca^2+^ channels and decreasing pericyte tone.^3^ Experiments on brain slices, where arterioles and capillaries tend to be isolated from each other by the slicing procedure, have reported a pharmacological distinction between activity-evoked dilations of penetrating arterioles and of capillaries.^37^ Arterioles are dilated by the release of NO, following activation of NO synthase (probably in interneurons) by synaptic glutamate release while, in contrast, pericyte-mediated capillary dilation results from activity-dependent ATP release from neurons acting on P2X receptors on astrocytes, raising astrocyte [Ca^2+^]_i_ and evoking release of PgE_2_. *In vivo*, where electrical coupling of endothelial cells and contractile mural cells means that hyperpolarization can spread between mural cells along capillary endothelial cells^14,47^ including probably also back to upstream arterioles,^48^ there could well be some mixing of these differing pharmacological profiles.

It has been pointed out that inhibiting the classical signalling pathways that have been proposed to mediate neurovascular coupling invariably blocks less than 70% of the increase of blood flow evoked by neuronal activity.^49^ It is likely that a neuronal activity-evoked increase of [K^+^]_o_ contributes at least part of the residual vessel dilation that increases cerebral blood flow, as K^+^ release from astrocyte Ca^2+^-gated K^+^ channels has been reported to hyperpolarize endothelial cells, vSMCs (and perhaps pericytes) by increasing the conductance of inward-rectifying K^+^ channels in the latter cells.^50,51^

In the heart, the changes of blood flow that are needed to power contraction in conditions of different heart rate are thought to be largely evoked by changes in the concentration of ‘metabolic messengers’ such as adenosine and K^+^ efflux from myocytes evoked by a fall of intracellular ATP concentration.^9,52^ Hyperpolarization of cardiac myocytes by K^+^ efflux may be transmitted to endothelial cells (and hence to gap junctionally connected pericytes) if there are gap junctions between the myocytes and the endothelial cells,^9^ or alternatively by K^+^ accumulation extracellularly hyperpolarizing endothelial cells by increasing the conductance of inward-rectifier channels in the latter cells. Sheer stress evoked release of vasodilating endothelial NO, and vasoconstriction by noradrenaline released from sympathetic nerves or circulating adrenaline, also regulate blood flow.^52^ These influences are likely to act on pericytes as well as on vSMCs.^11^

## 7. Pericytes constrict capillaries in ischaemia

During cerebral,^3,4^ cardiac,^11^ retinal,^14,53^ and renal^13^ ischaemia (in tissue slices, whole-mounted retinae or *in vivo* in rodents), focal microvascular constrictions appear at pericyte locations, suggesting that pericytes constrict capillaries and disrupt the microcirculation. Pericyte constriction is expected in ischaemia because removal (by cessation of blood flow) of the energy needed for ion pumping will lead to an inevitable increase of [Ca^2+^]_i_ (via ongoing Ca^2+^ entry through voltage-gated Ca^2+^ channels, and other entry pathways for Ca^2+^). This will lead to persistent constriction, as demonstrated using Ca^2+^-sensitive molecules in retinal pericytes.^53^ Calcium overload is facilitated by reactive oxygen species (ROS) production in cerebral ischaemia.^4,54,55^ Blocking voltage-gated Ca^2+^ channels or genetically knocking down α-SMA protein expression leads to a suppression of this constriction.^53^

## 8. Incomplete microcirculatory reflow after arterial recanalization

**Figure 3:**
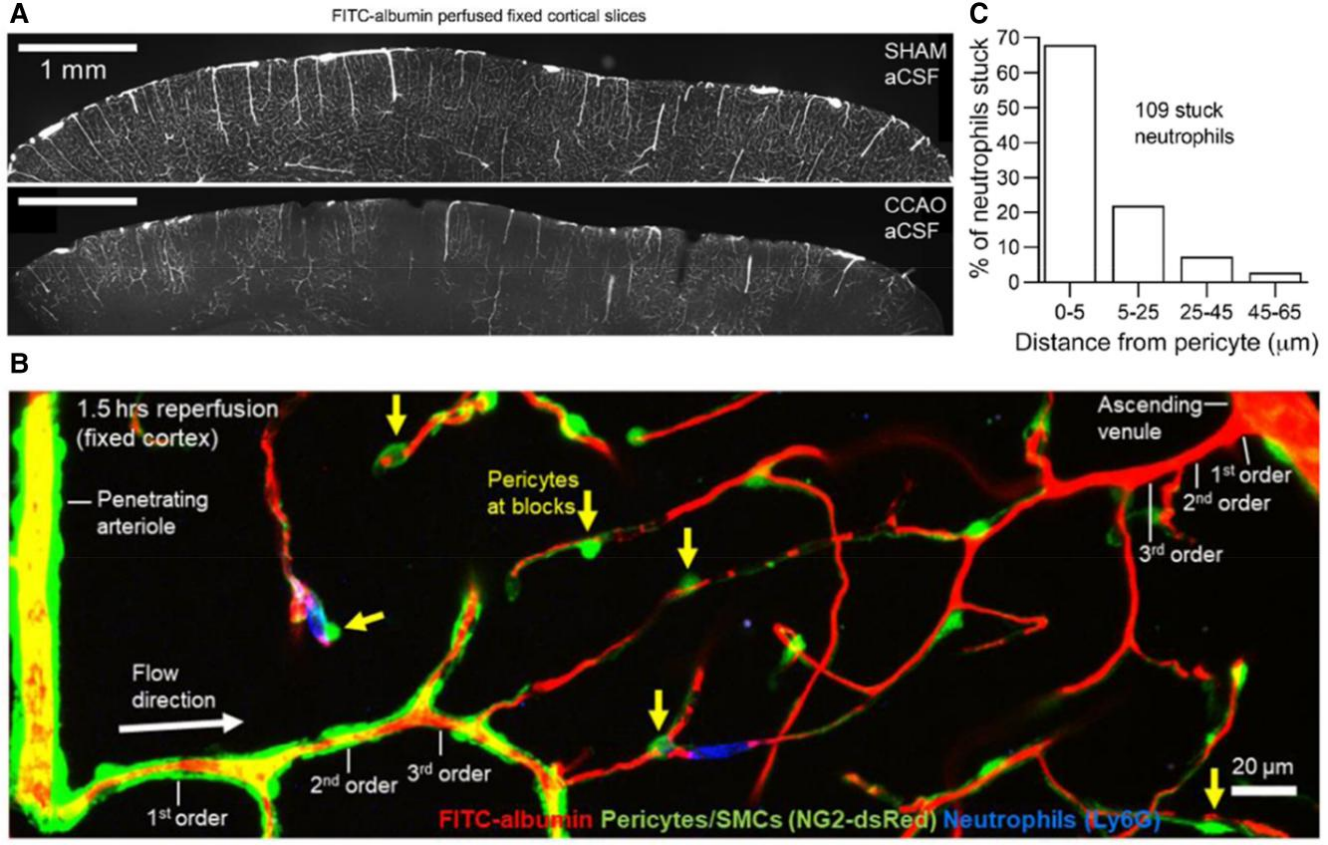
No-reflow and neutrophil blocks in cerebral cortex. Blood flow response to 1.5 h combined carotid artery occlusion (CCAO) and 1.5 h reperfusion *in vivo*. (*A*) FITC-albumin label in blood (white) shows complete perfusion of the microvasculature after a sham operation, but large areas of no-reflow after CCAO. (*B*) Vascular bed from penetrating arteriole (left) to ascending venule (right) with pericytes and vSMCs labelled green transgenically (NG2-dsRed recoloured) and with FITC-albumin in the blood (red, recoloured). Numerous locations are seen where pericytes are located close to blocks of flow (arrows), with neutrophils (labelled blue for Ly6G) stuck at the blocks. (*C*) Location of stuck neutrophils relative to nearest pericyte shows blocks are largely within a few microns of the pericyte, where most circumferential pericyte processes are located and capillary constriction is greatest.^5^ All figures from Korte *et al*.^34^

While it is unsurprising that pericytes constrict during ischaemia, it is far more surprising that they do not relax again after a period of ischaemia, i.e. on reperfusion that can be brought about clinically by removal of a thrombus, either using tissue plasminogen activator or using a stent-retriever. In all the tissues mentioned in the previous section, this leads to a failure to completely reperfuse the microvasculature, and ongoing tissue damage—the no-reflow phenomenon. In the brain, no-reflow (*Figure 3A*) was initially reported by Ames *et al*.^56^ after global ischaemia, and by Crowell and Olsson^57^ after focal cerebral ischaemia. After middle cerebral artery occlusion (MCAO), removal of the occlusion leads to a restoration of cerebral blood flow to only ∼50–80% of its original level (the magnitude depending on the severity and duration of the ischaemia). No-reflow in cardiac tissue will be dealt with in more detail below.

**Figure 4:**
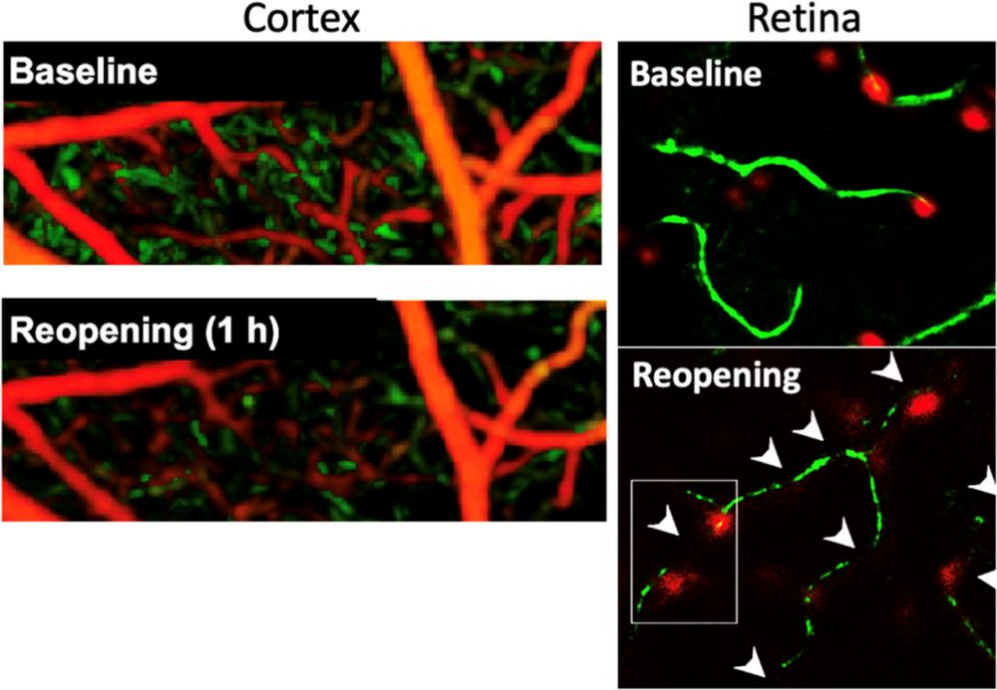
*In vivo* imaging of no-reflow by detecting erythrocyte stalls. Monitoring erythrocyte motion (green) allows imaging of blood flow and can depict RBC stalls, and hence no-reflow, with higher fidelity than imaging plasma flow. Left images are maximum intensity projections of OCT angiograms from mouse cortex before and 1 h after 2 h MCA occlusion, showing incomplete reperfusion of capillaries deep in cortex (pseudo-coloured green; surface vessels were pseudo-coloured red). Right images are adaptive optics scanning light ophthalmoscopy (AOSLO) images of the retina of an NG2-dsRed mouse before and after 1 h central retinal artery occlusion. In contrast to imaging the brain, which often necessitates invasive surgery, imaging the retina can be achieved non-invasively through the lens, minimizing potential experimental confounding effects. After recanalization, the microcirculation could not be visualized in some microvessels (no-reflow) because vessels become visible only if there is erythrocyte motion in their lumen (pseudo-coloured green), while some microvessels exhibit a thin stream of flow (incomplete reperfusion). The frequent RBC stalls (black segments, arrowheads) are caused by capillary constrictions, at some of which red fluorescent pericyte somata can be seen. The figures were adapted from Alarcon-Martinez *et al.*^53^ and Lee *et al.*^61^ (© Optical Society of America) with permission. The contrast was adjusted to accentuate colours.

**Figure 5:**
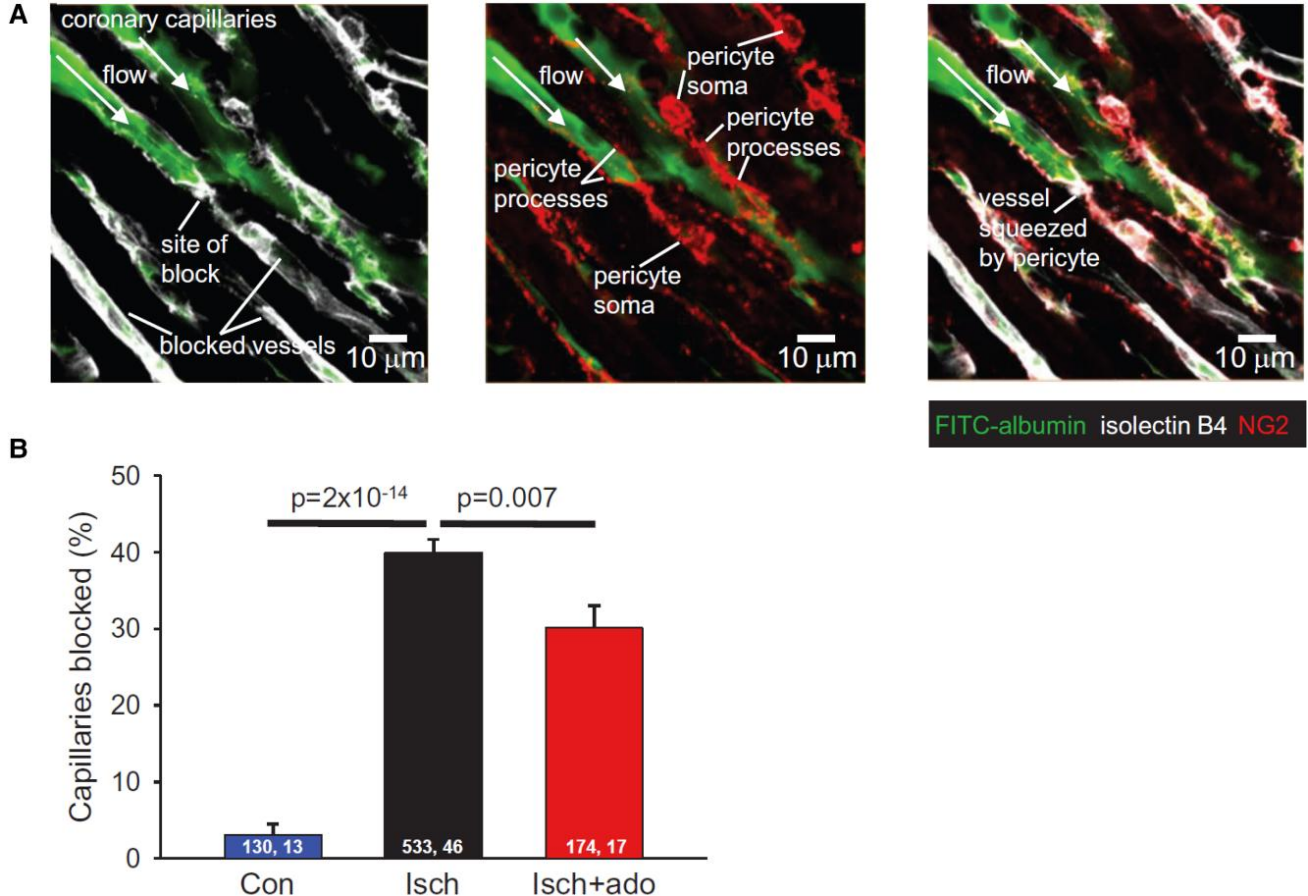
No-reflow and capillary block after ischaemia in myocardial capillaries. (*A*) Images of rat left ventricular capillaries labelled for basement membrane (IB4, white), pericytes (NG2, red) and with FITC-albumin in the blood 15 min after a 45 min period of occlusion of the left anterior descending coronary artery (LAD). Some capillaries are reperfused, others are not, and the blocks of the capillaries tend to occur near pericytes. (*B*) Percentage of capillaries blocked after a sham operation, after LAD occlusion and reperfusion, and after LAD block and reperfusion with injection of adenosine. Numbers on bars are numbers of capillaries examined, image stacks examined from five sham, six ischaemic, and eight ischaemic plus adenosine hearts. Images and graph are from O'Farrell *et al*.^11^

Clots formed by leucocytes, platelets, and fibrin have been suggested as potential sources of the no-reflow phenomenon.^58–60^ However, recent studies have revealed that, in the CNS^3,4,53^ (*Figures 3B* and *C* and *4*), heart^11^ (*Figure 5*), and kidney,^13^ constriction of capillaries by pericytes is a major cause of no-reflow, and is in fact the cause of the presence of leucocytes, platelets, and red blood cells lodging at the loci of capillary block (*Figures 3B* and *C* and *4*).^11,34^ The relative frequency of blocks by different cell types will depend on how constricted the capillaries are, the larger size and rigidity of neutrophils relative to other cell types, and the occurrence of cell adhesion molecules on the endothelial cells to facilitate entrapment of the cells. Cell accumulation and endothelial damage promote fibrin formation, resulting in strengthening of clots at constricted capillary sites. Importantly, the spatially localized nature of constrictions at pericytes, and the associated presence of trapped cells within a few microns of these sites,^11,34^ conform with an active contraction rather than generalized capillary compression by diffuse cellular swellings of astrocyte endfeet or endothelial cells.

However, even cell-mediated occlusion of capillaries would be expected to be reversed if pericyte-mediated constriction is reversed on reperfusion of upstream arteries. So why does this not occur? After a period of severe ischaemia, applying extracellular propidium ions, which are normally excluded from cells because of propidium’s 3+ charge but which fluoresce on binding to DNA if they can enter the cell, reveals that most pericytes suffer a profound loss of membrane integrity and thus appear to die.^3^ So ischaemia first triggers pericyte contraction and capillary constriction (leading to occlusion of some capillary segments by neutrophils and other cells), but then kills the pericytes while they are in contractile rigour. The pericytes cannot relax after this to allow capillary dilation because of the absence of ATP. Thus, pericytes may produce a tight lasso around capillaries, which will only be removed when immune cells remove the whole cell. The time needed for pericyte death is relatively brief (1 h^3^) in brain slice experiments inhibiting ATP production pharmacologically (mimicking very severe ischaemia) but takes hours^4,61^ in *in vivo* experiments using MCAO. In the CNS, this injury and death of pericytes, along with factors like matrix metalloproteinase activation, may contribute to the loss of blood–brain or blood–retina barrier integrity, as observed in pericyte-deficient mice,^24–26^ which may be restored (and angiogenesis increased), by transplantation of hPSC-derived pericyte-like cells,^62^ or by blocking Nogo-A signalling to restore pericyte coverage.^63^

Sophisticated imaging techniques utilized in animal experiments can distinguish impaired reflow at the artery and capillary levels. However, routine clinical imaging techniques have limitations in this regard, as discussed below, and can only detect blockages in the larger vessels. The application of optical coherence tomography angiography to the retina, with technological improvements, may finally allow resolution of post-ischaemic pericyte-mediated capillary blocks in routine clinical imaging.^53,61^

The following two sections consider pericyte-mediated no-reflow in the brain and in the heart in more detail.

## 9. No-reflow in human stroke and its imaging

For patients with acute ischaemic stroke caused by an anterior circulation large vessel occlusion, intravenous thrombolysis followed by endovascular thrombectomy has become the standard of care. Although this treatment achieves ‘successful revascularization’ (defined below) in more than 70% of large vessel occlusion patients, at least half of these do not return to independent living after their stroke^64^—referred to as futile reperfusion. No-reflow is a likely contributor to futile reperfusion and readers are referred to two excellent, recent reviews on the discovery of the no-reflow phenomenon in animal models and the challenges facing the identification and characterization of no-reflow in patients.^65,66^

The success of revascularization therapy can be assessed by comparing 3D angiography images acquired before and immediately after treatment. Using versions of the Thrombolysis in Cerebral Infarction (TICI) scale,^67,68^ treating physicians and researchers can thus quantify the percentage of the vascular territory downstream of the large vessel occlusion that becomes perfused as a result of the treatment. Accordingly, successful revascularization is defined as the revascularization of more than 50% of the vascular territory affected by the stroke^64^ whereas complete revascularization (90–100% of the affected vascular territory) is achieved in 32% of patients.^68^ In terms of vascular reflow, revascularization *per se* is hence unsuccessful or incomplete in 68% of acute ischaemic stroke patients with anterior circulation large vessel occlusion, despite state-of-the-art treatment.

The extent of tissue damage after acute ischaemic stroke is attributed to the severity of the accompanying reduction in cerebral blood flow (cerebral blood flow, measured in mL blood per 100 mL tissue per minute). The ischaemic threshold is hence defined as a cerebral blood flow threshold, below which neurological symptoms emerge and irreversible tissue injury (infarction) is imminent unless cerebral blood flow is restored.^69^ Such critically hypoperfused, yet un-infarcted tissue, is referred to as the ischaemic penumbra and is the target of acute ischaemic stroke treatment, in that salvageable tissue may remain as long as 24 h after symptom onset. In practice, critical hypoperfusion is typically determined from acute magnetic resonance or computerized tomography based perfusion imaging. Dynamic images are acquired during the passage of bolus-injected contrast medium, and post-processed to yield parametric maps of cerebral blood flow, cerebral blood volume, blood mean transit time (MTT), and Tmax, a transit-time related index of tissue contrast medium retention. A Tmax > 6 s is often used to define critical tissue hypoperfusion as part of acute ischaemic stroke management.

To address the existence and significance of the no-reflow phenomenon, several studies have examined acute ischaemic stroke patients with large vessel occlusion in whom complete revascularization was achieved—in order to identify any persistent, critically hypoperfused tissue.^70^ Defining no-reflow as the presence of tissue with Tmax > 6 s despite 100% angiographic reperfusion (TICI score 3), residual, critically hypoperfused tissue was found in 43% of patients within 30 min of recanalization.^71^ Similarly, searching for regions with visibly reduced perfusion (and requiring that cerebral blood flow and volume values were ≥15% lower than those of contralateral mirror regions), in acute ischaemic stroke patients 24 h after complete (90–100%) revascularization was achieved, no-reflow tissue was found in 25% of patients, encompassing 60% of their infarct volumes.^72^ The presence of no-reflow tissue, in turn, was associated with post-treatment complications and poor outcome (dependence or death) after 3 months.^72^ The prevalence of persistent hypoperfusion and its attribution to no-reflow in patients with angiography-proven complete reperfusion remains debated, ranging from 13–43% in most studies.^70^ Similarly, using arterial spin labelling to measure cerebral blood flow a prevalence of no-reflow after stroke of 24% was found,^73^ while using Single-Photon Emission Tomography cerebral blood flow measurements found hypoperfusion in 25% of patients.^74^ Proposing that no-reflow leads to increased microvascular resistance post-recanalization, and using the pulsatility index, as determined by transcranial Doppler in upstream arteries, to detect the phenomenon, gave a prevalence of 28%.^75^

**Figure 6:**
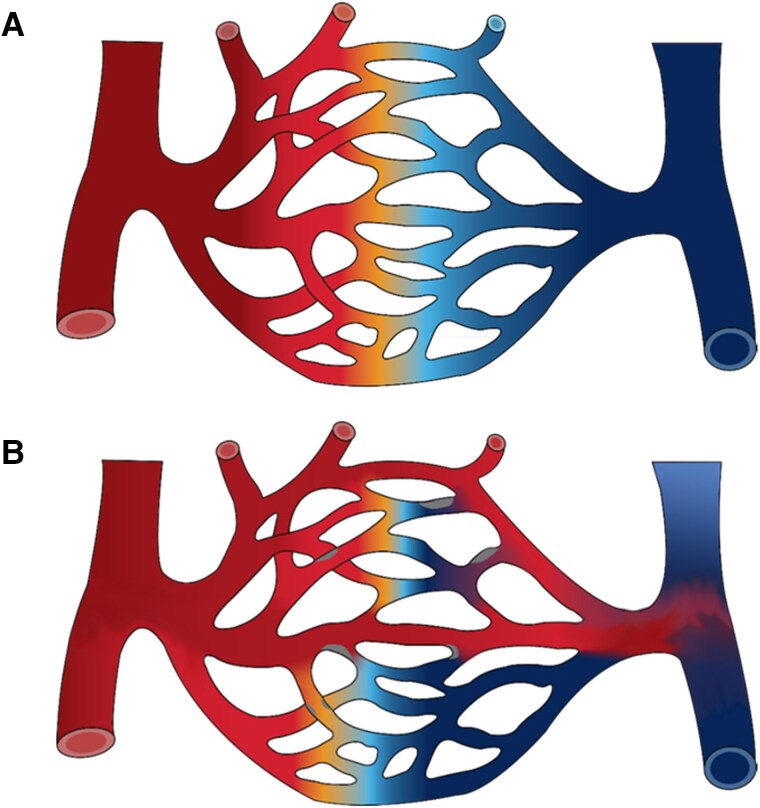
Capillary transit-time heterogeneity reduces oxygen extraction. The figure illustrates the passage of fully oxygenated blood (indicated as dark red) through a capillary bed, during which some of blood’s oxygen is extracted by the surrounding tissue (indicated by the gradual shift towards lighter red, orange, and darker blue colours from left to right). (*A*) The widely held misconception that oxygen availability can be inferred solely from tissue blood flow (measured in mL blood per 100 mL tissue per minute) rests on the assumption that blood flow is uniformly distributed across the capillary bed, such that identical fractions of the blood’s oxygen content is extracted from individual capillary paths.^69^ This idealized condition of negligible capillary transit-time heterogeneity provides the most effective extraction of blood’s oxygen content but is not typical of normal tissue. (*B*) This panel illustrates how capillary transit-time heterogeneity reduces net oxygen extraction compared to panel *A*, although blood flow is identical in the two conditions. This ‘shunting’ effect arises when the time available for blood-to-tissue oxygen extraction (before blood returns to the heart) becomes too short—e.g. due to constrictions or occlusions along some capillary paths.

The ‘macroscopic’ perfusion indices described above may not capture the pathophysiological significance of impaired microvascular reperfusion, however. Specifically, the assumption that critical hypoperfusion can be characterized solely in terms of cerebral blood flow or related perfusion thresholds was recently challenged,^76^ because tissue oxygen availability depends on both cerebral blood flow *and* the microscopic distribution of the blood.^77^ This dependency arises from the fact that extraction of oxygen from individual capillaries by the tissue is limited by the blood’s capillary transit time: the time available for blood-tissue diffusion exchange before blood returns to the heart (see *Figure 6*).

Based on this limitation, the impact of per-ischaemic capillary no-flow and subsequent capillary no-reflow, respectively, on overall tissue oxygenation can be illustrated as follows. Imagine that tissue cerebral blood flow suddenly falls from 50 to 20 mL/100 mL/min because of a large vessel occlusion—while cellular oxygen utilization remains unchanged, sustained by complete (100%) extraction of blood’s oxygen content. Now, imagine that 50% of tissue capillaries close due to pericyte-mediated changes (as reviewed above) without affecting residual blood flow. This reduces capillary transit times (in the capillaries remaining open that now pass twice as much blood) by as much as 50% and therefore reduces the percentage of blood oxygen content being extracted—significantly increasing the risk of hypoxic tissue injury. Next, imagine that recanalization normalizes cerebral blood flow while 50% of capillaries remain closed (no-reflow). Transit times will then be half of that in normal tissue. Oxygen extraction is therefore impaired and oxygen availability reduced, although cerebral blood flow is restored.

These examples illustrate three central, pathophysiological implications of the ischaemia-related microvascular changes described in the previous sections: (i) tissue oxygenation cannot be gleaned from blood flow in the presence of per-ischaemic capillary no-flow, nor no-reflow after revascularization, (ii) capillary no-flow accelerates hypoxic tissue injury (loss of salvageable tissue) during ischaemia, irrespective of its effects on residual blood flow, and (iii) after revascularization, capillary no-reflow reduces tissue oxygen availability compared to similar tissue with capillary reflow—and may continue to perpetuate hypoxic tissue injury although ‘macroscopic’ perfusion is normal or even elevated.^76,78^

The microscopic distribution of blood (or rather: plasma, as the methods rely on intravascular, extracellular contrast medium) can be derived from data acquired with the perfusion imaging methods described above, applying post-processing algorithms that include the heterogeneity (variance) of blood’s capillary flows (or transit times) as an additional parameter.^79,80^ Applying these algorithms to perfusion data acquired in acute ischaemic stroke patients, it turns out that altered microvascular flow (or transit time) heterogeneity at the time of acute perfusion imaging (typically within 3–12 h of symptom onset) is a better predictor than low cerebral blood flow of subsequent infarction^81–84^ in patients where revascularization is not achieved (permanent ischaemia). These findings contradict the notion that cerebral blood flow alone determines the extent of hypoxic tissue injury after stroke, suggesting that progressive microvascular flow disturbances gradually reduce oxygen availability in hypoperfused tissue until blood flow is restored. Importantly, early reperfusion in acute ischemic stroke patients appears to normalize blood’s microvascular distribution throughout most of the tissue affected by ischaemia.^84^

The spatial resolution of human perfusion images is on the order of millimetres and each image voxel therefore contains tens of thousands of capillary segments. Simulation studies show that the changes in the heterogeneity of blood’s intravoxel distribution during stroke are consistent with no-flow through capillary paths with high resistance.^84^ These pathways are the most susceptible to no-flow during episodes of low perfusion pressure and also tend to represent the longest capillary pathways, giving the appearance of microvascular flow homogenizations.^84^ This interpretation is consistent with the pericyte-mediated capillary-level mechanisms reviewed above and with direct observations of capillary flow dynamics in rodent stroke.^85^ Capillary no-flow would be expected to reduce local cerebral blood volume, but human perfusion imaging data showed only marginal blood volume reductions in areas with intravoxel flow homogenization.^84^ Capillary no-flow is likely to cause local hypoxia and elicit compensatory dilation of remaining (open) capillaries, which could contribute to this finding.

Interestingly, simulation studies show that the Tmax parameter obtained by perfusion imaging has an unintended sensitivity to combinations of long MTT and low microvascular heterogeneity^76^—and hence to the relative homogenization of microvascular flows observed in hypoperfused tissue after acute ischaemic stroke.^84^ This coincidental bias may explain why studies of no-reflow after revascularization have found significant Tmax abnormalities despite seemingly normal cerebral blood flow and volume values.^71^ A relation between elevated Tmax and relative homogenization of microvascular flows was indeed reported in a recent study^86^ that used the perfusion algorithm described above to disentangle macro- and microvascular flow disturbances before and immediately after endovascular treatment, and to calculate the corresponding oxygen extraction based on its biophysical relation to blood flow and blood’s microscopic distribution.^77^

Taken together, clinical studies support the presence and clinical significance of microvascular no-reflow in acute ischaemic stroke, but methodological challenges remain in terms of diagnosing and characterizing this phenomenon. Specifically, better understanding of the relations between microvascular no-reflow, blood flow, and tissue oxygen availability is needed in order to fully understand the pathophysiology of hypoxic tissue injury during ischaemia and after revascularization therapy. Thus, while macrovascular no-reflow (negligible cerebral blood flow) after revascularization is clearly deleterious, biophysical models further predict that hypoxia may persist—and even become worse—after cerebral blood flow is normalized, if blood’s microvascular distribution is not restored in parallel.^76,77,87^ Future studies should therefore examine the putative roles of microvascular no-reflow in cases where tissue infarction and poor outcomes are observed despite normal or even high cerebral blood flow (cf. reports^78,87,88^ of ‘reperfusion injury’ and ‘luxury perfusion’). Thus far, contributions of reperfusion to irreversible tissue injury have mainly been reported in the myocardium (see below).

The microcirculation’s impact on tissue oxygenation implies that per-ischaemic no-flow and post-revascularization no-reflow should be considered simultaneously as we try to understand acute ischaemic stroke patient outcomes, as the former could place individual patients on a trajectory of rapid infarct growth although revascularization is achieved. These considerations could also include patients’ pre-existing vascular risk factor as these are likely to render the microcirculation more vulnerable to ischaemic injury—and also extend to outcomes beyond functional recovery at 3 months, as stroke and microvascular dysfunction are increasingly being linked to cognitive decline and dementia.^89,90^

## 10. Coronary microvascular injury from myocardial ischaemia/reperfusion

Whereas the pathophysiology of acute myocardial infarction (AMI) has traditionally focused on cardiomyocyte necrosis resulting from ischaemia, a number of paradigmatic changes in the view on AMI have emerged in the last few decades. Reperfusion, which was traditionally viewed only as the salvage from ischaemia, was demonstrated to induce contractile dysfunction even after reversible myocardial ischaemia (stunning).^91,92^ The contribution of reperfusion to irreversible injury was long debated^93^ but ultimately appreciated with the recognition of ischaemic post-conditioning, an intervention that reduced infarct size even when performed during early reperfusion.^94^ Since then, AMI has been viewed as a result of myocardial ischaemia/reperfusion injury.^95,96^ Novel modes of cell death (other than necrosis) have been recognized to contribute to AMI.^96,97^ Finally, the heart is a multi-cellular organ^98^ and not only cardiomyocytes but also a number of other cellular compartments are involved in AMI.

The coronary circulation plays a particularly prominent role in AMI, because it is both, the culprit and a victim of AMI.^99–101^ Plaque rupture^102,103^ and plaque erosion^104,105^ initiate myocardial ischaemia and AMI. However, re-opening of the occluded coronary artery to terminate ischaemia often does not restore coronary blood flow to the microvasculature. Such coronary no-reflow was recognized in 1966 by Krug *et al*.^106^ but received wider attention when confirmed and characterized in more detail by Kloner *et al*.^107^ in 1974. With the advent of interventional coronary reperfusion^108^ for treating AMI, the no-reflow phenomenon became a frequently observed clinical entity.^109,110^ No-reflow following interventional reperfusion carries an adverse prognosis, apart from and beyond that of the infarct size.^111–113^

The mechanisms proposed to underlie reperfusion injury to the coronary microcirculation that result in slow or no-reflow are multifold and include^99–101,110^: (i) embolization of plaque debris and thrombotic material that physically obstruct the microcirculation^114^; (ii) active vasoconstriction as a result of sympathetic reflex activation^115^ with resulting α-adrenergic vasoconstriction^116^; (iii) endothelial dysfunction and vasoconstriction in response to TNFα, serotonin, thromboxane, and endothelin released from the stented lesion^117,118^; (iv) platelet- and platelet–leucocyte aggregates^119–121^; (v) erythrocyte stasis^122^; (vi) compression by extravascular edema^123^; and (vii) structural damage to the capillary endothelium with shedding of endothelial cells into the lumen^107^ and capillary rupture with subsequent bleeding into the interstitial space.^124,125^ To these mechanisms, a novel proposal^126^ was recently added i.e. that, by analogy with cerebral post-ischaemic no-reflow, coronary capillary constriction by pericyte contraction could be a major contributor to the no-reflow phenomenon, and to block of capillaries by neutrophils, erythrocytes, and platelets. This was validated experimentally,^11,12^ and a reduction of pericyte-mediated capillary constriction has also been demonstrated to underlie ischaemic preconditioning evoked myocardial protection.^127^ In retrospect, with the recognition of the role of pericytes in coronary microvascular perfusion during myocardial ischaemia/reperfusion, it is possible that the alpha_2_-coronary vasoconstriction that contributed to myocardial ischaemia in dogs^128,129^ and patients^116,130^ was partly generated by pericytes.^36^

It could be proposed that smooth muscle and pericyte contraction are not purely deleterious, but might also be protective against capillary rupture and intramyocardial haemorrhage, i.e. the most fatal manifestation of myocardial ischaemia/reperfusion injury. Once pharmacological therapies to prevent coronary pericyte contraction after ischaemia have been developed (see below), it will be interesting to test whether the overall outcome after cardiac ischaemia is improved by blocking ischaemia-evoked pericyte contraction.

In the clinical situation of interventional reperfusion for AMI, coronary microvascular injury is apparent from the slow/no run-off of the contrast medium during angiography that can be semi-quantitatively graded by a TIMI (Thrombolysis in Myocardial Infarction) score or TIMI frame count. Also, myocardial perfusion can be graded from the blush of the contrast medium.^99^ A more sophisticated and comprehensive assessment of the coronary microcirculation is obtained from measurement of coronary flow velocity (Doppler) or flow (thermodilution) along with perfusion pressure and the calculation of coronary microvascular resistance,^131–133^ but such measurement is not routine and is reserved for academic studies. The gold standard, not only for the assessment of infarct size and global and regional left ventricular function but also of coronary microvascular obstruction and haemorrhage, is cardiac magnetic resonance imaging (CMR).^134^ The advantage of CMR is its non-invasive nature that permits serial measurements, but the disadvantage is its restricted availability that usually permits measurements only days after the procedure.

In CMR, infarct size and coronary microvascular obstruction go often, but not always in parallel.^100^ CMR has also re-identified intramyocardial haemorrhage as the most severe manifestation of coronary microvascular injury^135–138^; haemorrhage had been reported in experimental studies in the 1980s^124,125^ but thereafter was only considered as a complication of thrombolysis.^139^ Intramyocardial haemorrhage promotes inflammation and adverse remodelling,^140^ and haemorrhage on CMR carries additional prognostic information beyond that given by infarct size in patients with AMI.^137^

A specific protection from coronary microvascular injury is not available^141^; there is only a single preclinical study that reported primary coronary microvascular protection^142^ in a mouse model of AMI by angiopoietin-like peptide 4. Other than that, intracoronary calcium antagonists and/or nitroprusside are used clinically to treat coronary no-reflow, with variable success.^143^ Given the importance of coronary microvascular injury in AMI, the most recent Canadian Cardiovascular Society classification graded AMI classes (1) and (2) as minor and significant cardiomyocyte necrosis without coronary microvascular injury, but (3) as cardiomyocyte necrosis with coronary microvascular obstruction and (4) as cardiomyocyte necrosis with intramyocardial haemorrhage.^144^

Pericytes, apart from their contribution to the acute coronary microvascular injury, have been suggested to mediate the fibrotic response after ischaemia^145^ (although, as discussed for the brain above, this conclusion may be confounded by the presence of perivascular fibroblasts that also express *Cspg4*/NG2). Transplantation of pericytes may also reduce the formation of fibrosis and promote angiogenesis.^146^

## 11. Therapeutic approaches targeting pericytes

We have described above how, after ischaemia in the brain and heart, blood flow remains significantly depressed (by up to 50%) as a result of microvascular constrictions generated at pericyte locations. It seems an obvious therapeutic approach, therefore, to enhance post-ischaemic blood flow by reducing pericyte contraction. There are already numerous examples of the successful application of this idea in different animal tissues.

Pioneering work in the brain established that prevention of oxidative–nitrative stress just before reperfusion decreased the percentage of capillaries that constricted during ischaemia, and provided neuroprotection, presumably by increasing cerebral blood flow.^4,147^ Intravitreally administered amlodipine (a voltage-gated calcium channel blocker), adenosine, and the gap junction blocker carbenoxolone also prevented ischaemia-induced capillary constrictions in the retina^53^ (a region of the CNS that can be imaged non-invasively through the eye). Similar results have been obtained by the Attwell (UCL) and Buchan (Oxford) groups (unpublished) when injecting the voltage-gated calcium channel blocker nimodipine on reperfusion after MCAO in rats, and C-type natriuretic peptide has been shown to prevent pericyte constriction evoked by amyloid beta and ROS evoked release of endothelin.^5^ Thus, impaired reperfusion can be reversed before pericytes die.

Similarly, in the heart, administration of intravenous adenosine just before reperfusion after occlusion of the left anterior descending coronary artery led to a reduction of pericyte-mediated capillary constriction, and an increase of coronary blood volume.^11^ In studies of this sort, demonstrating a reduction of pericyte-mediated constriction establishes that the drug applied is operating at least in part by relaxing pericytes (although relaxation of arteriolar SMCs may also contribute). Interestingly, adenosine attenuated coronary microvascular obstruction in a number of trials in patients with reperfused acute myocardial infarction, but did not improve clinical outcome (although it was acknowledged that a larger study was needed).^148^ Adenosine will tend to dilate vessels throughout the body and lower blood pressure (although autoregulation opposes this) so, in a study on brain ischaemia, systemic administration of adenosine-squalene nanoparticles was used to improve cerebral microcirculation after recanalization in mouse stroke models, with the nanoparticle formulation reducing the hypotensive impact of adenosine upon systemic administration, potentially due to its enhanced capture by endothelial cells in areas with sluggish flow.^149^ Finally, in an insight gained from the kidney that may be relevant to the brain and heart, administration of the clinically used Rho kinase blocker hydroxyfasudil from the start of reperfusion, after a period of occluding the renal artery, led to dilation of capillaries in the renal cortex and medulla, to increased blood volume and to a reduction of kidney injury.^13^

## 12. Concluding remarks

One dominant manner in which pericytes cause clinically relevant tissue damage is by constricting capillaries and reducing blood flow. It seems likely that developing strategies to block pericyte-mediated capillary constriction could significantly improve clinical outcome following ischaemia, and possibly also in the decrease of blood flow occurring in Alzheimer’s disease and COVID-19 infection. In the brain, pericyte loss also causes loss of BBB function (it would be of interest to know how pericyte loss affects the permeability of coronary capillaries). Remarkably, there seems to be no clear understanding of whether the loss of BBB function is secondary to a loss of blood flow, whether blood flow falls following a loss of BBB function, or whether these events are independent. Establishing causal links between these events would likely inform us whether blood flow reduction, BBB dysfunction, or both are the best target for therapy. A further crucial issue is our limited knowledge of how pericyte function varies with position in the capillary bed, especially in the heart. While the first few branches of the capillary network in the brain have both the highest resistance and the greatest contractile power, and so might be the most important target for drugs that reduce contraction, downstream pericytes also constrict capillaries and may have different mechanisms controlling [Ca^2+^]_i_, implying different drug targets for reducing contraction. At present, it is unclear how pericyte function varies with position along the arteriovenous axis in the heart.

Despite these uncertainties, overall, the future looks bright for the development of drugs that could either prevent post-ischaemic no-reflow by inhibiting the constriction of capillaries by pericytes, or (in the brain) prevent BBB dysfunction that results from pericyte loss.
